# Epidemiology, Clinical Microbiology and Antimicrobial Therapy: A Shared Effort Against Infectious Diseases

**DOI:** 10.3390/antibiotics15010097

**Published:** 2026-01-17

**Authors:** Francesca Pica

**Affiliations:** Department of Experimental Medicine, University of Rome Tor Vergata, 00133 Rome, Italy; pica@uniroma2.it

The last few years have confirmed that infectious diseases are characterized not only by new emerging threats (i.e., avian influenza and MPox), but also by continued concerns over healthcare-associated infections (HAIs), antimicrobial resistance (AMR) and the resurgence of vaccine-preventable diseases [1]. A recent WHO report presents a global analysis of AMR prevalence and trends based on more than 23 million bacteriologically confirmed cases of bloodstream infections, urinary tract infections, gastrointestinal infections and urogenital gonorrhea (from 104 countries in 2023 and 110 countries in the period 2016–2022) [1]. The analysis of 93 infection type–pathogen–antibiotic combinations indicates a global pattern of AMR characterized by widespread resistance to essential first-choice, second-choice and last resort antibiotics, although with variation among pathogens and countries due to climate and socioeconomic factors [2].

Crucial to managing AMR is identifying mutations that lead to resistance in a given genomic and environmental background. Today there are new techniques that enable quantitative comparison of which genes are under antibiotic selection and capture how genetic background influences resistance evolution [3]. Several reports also highlight the importance of in vitro testing of the evolution and mechanisms underlying resistance with complementary methods and in multiple relevant bacterial species [4]. The progress in this field will provide us with further useful elements in the fight against infectious diseases and AMR, but there is a need for a strong collaborative network among epidemiologists, clinicians, clinical microbiologists, pharmacologists, bioinformaticians and basic-science researchers to fully understand what is really happening and what we can do. This Special Issue has collected information that can help shed light on interesting aspects in this area.

The first piece of information can be inferred by three studies (Contributions 1–3), performed in Romania and in Italy in a post-COVID-19 period, that show the consequences of missed or incomplete *Bordetella pertussis* vaccinations, leading to a resurgence of pediatric cases. Most affected children were under 3 years of age and some under two months (pre-vaccination era). The clinic of disease requested hospitalization and most severe cases exhibited hyperleukocytosis associated with complications such as heart failure. A significant correlation was observed among intensity of the lung radiological picture, WBC count and CRP, with a more severe picture in the unvaccinated or partially vaccinated subjects. Moreover, the impact of other bacterial (mainly *S. pneumoniae* and *H. influenzae*, but also *M. pneumoniae* and *P. jirovecii*) and viral [enteroviruses (EV-HRV) and SARS-CoV-2, Human Parainfluenza Virus-3 (HPIV-3), Human Adenovirus (HAdVs), etc.] co-infections on the clinical outcome was analyzed. Interestingly, many cases were initially misdiagnosed as viral respiratory infections, with delays in appropriate treatment and contact tracing. This is not a trivial point, considering that a combined antibiotic and antiviral therapy may be indicated in very young and/or fragile subjects. Moreover, although qPCR testing has enhanced early detection, its sensitivity diminishes as the disease progresses, particularly in later stages. Thus, an accurate and timely diagnosis of pertussis still remains a significant challenge, as reported in the literature [5,6,7,8,9]. The easing of COVID-19 pandemic restrictions played a role in the subsequent increase in cases, but other factors also contributed to this trend, such as insufficient access to healthcare in rural areas, inconsistent public health messaging and growing vaccine hesitancy fueled by misinformation. The authors conclude that it is mandatory to pay increased attention to clinical and laboratory diagnosis, to acknowledge the emerging AMR of microorganisms circulating in local contexts and to implement vaccination of children and pregnant women. Similar issues and concerns have been raised by other research groups worldwide [10,11,12]. Moreover, considering the first-choice antibiotics used in this infection, it is important to consider that AMR against azithromycin has been found increasing globally, so that the WHO has classified azithromycin as an “antibiotic to watch” [13,14].

Contribution 4 addresses another important point, namely access to essential medicines in local contexts, noting that most antimicrobial stewardship (AMS) interventions focus on the appropriateness of indications, dosages and duration of treatment, on the implementation of measures aimed at reducing inappropriate prescriptions, and on improving knowledge of drugs, often neglecting the crucial role of access to essential medicines in achieving these results. Starting from the results of a previous study in the Campania Region (Southern Italy) that confirmed the effectiveness of a multifaceted antimicrobial stewardship program in reducing prescription rates and use of broad-spectrum molecules in the primary care setting [15,16], the authors show that since autumn 2022, the amoxicillin shortage was reported at a national level, with two practical consequences: (i) respiratory pathogens resurged in children after the easing of COVID-19 pandemic restrictions and (ii) the lack of amoxicillin led to increased prescriptions of second-line antibiotics (amoxicillin-clavulanate and third-generation cephalosporins). This resulted in a decline of the amoxicillin/amoxicillin–clavulanate index in Italian regions, reverting the effect of the previous successful stewardship measures. Of note, an amoxicillin shortage has been described in several Western countries in recent years [17]. The authors therefore suggest that these findings should prompt authorities and stakeholders to implement interventions to ensure the availability of essential medicines in communities through appropriate economic and health policies, in addition to raising public awareness of the prudent use of antibiotics.

Contribution 5 reports the results of a two-year, retrospective, single-center observational study at the internal medicine department (IMD) of a regional specialist hospital, the Centre of Pulmonology and Thoracic Surgery in Bystra, Poland. The authors focused on peripheral intravenous therapy procedures, including vascular access characteristics and related complications, as well as qualitative and quantitative analyses of drug consumption. They report that within the considered IMD, most patients required the use of a Peripheral Intravenous Catheter (PIVC) and antibiotics dominated the group of drugs administered intravenously. Of note, during the COVID-19 pandemic, three-quarters of the patients received antibiotics, with a much lower percentage of bacterial co-infections [18]. It is known that a properly functioning PIVC is crucial for antibiotic treatment and that, according to the recommendations, a single PIVC should provide vascular access for approximately 7 days of intravenous therapy; but the results of the study have shown that around twenty percent of PIVC were lost within the first 24 h after their insertion, mainly for complications and in elderly patients. This was attributed in part to decreased cognitive function of geriatric population, which prevents proper communication with the medical staff, but also to positioning errors due to the difficulty of finding adequate venous access. Nothing is trivial when aiming for successful antibiotic therapy, therefore the responsibility of the nursing staff to choose the appropriate place for vascular access device insertion and to take care of the vascular line is emphasized.

Complex but very interesting is Contribution 6, which explores the relationship between exposure of the microbiome to various antibiotic regimens and the risk of blood stream infections (BSIs) using a tool known as structural equation modelling (SEM) [19]. SEM enables competing theoretical causation networks to be tested all together by comparison with data from the literature. These causation models have three conceptual steps: exposure to specific antimicrobials are the key drivers, clinically relevant infection end points are the measurable observables, and the activity of key microbiome constituents on microbial invasion serve as mediators. These mediators, which may serve to promote, to impede, or to have no effect, are normally unobservable and are considered as latent variables in each model. The comparisons performed in the study used data from 200 to 300 studies that reported pneumonia and BSIs via several “alert” microorganisms in ICU patients, mostly receiving prolonged mechanical ventilation. By these comparisons, exposing ICU patients to topical antibiotic prophylaxis emerges as harmful in this context through its effects mediated by concurrency and Candida colonization. Although these data are unequivocal, it is currently difficult to predict their possible practical therapeutic implications in ICUs. However, these studies highlight the complexity of microorganism/host and resident microbiota/exogenous pathogen interactions and prompt us to investigate them further.

Contribution 7 describe a Gram-positive, strictly anaerobic, spore-forming, rod-shaped bacterium belonging to the phylum Firmicutes and the family Lachnospiraceae, first isolated about twenty years ago, i.e., *Robinsoniella peorensis*. By using the narrative review method, the authors report its epidemiological, clinical, and microbiological characteristics and provide information about its AMR, treatment and outcomes. This infection is rare and targets mostly joints and bones with a generally favorable prognosis, but it can cause lethal bacteremia in oncologic patients undergoing chemotherapy. The identification of novel microorganisms is more frequent in the current era, since genetic methods such as 16S rRNA gene sequencing have been implemented [20,21], making it possible to diagnose infections caused by microorganisms that are harder to identify by classic microbiological means [22].

There is then a group of manuscripts (Contributions 8–12) that specifically deal with antibiotic-resistant bacteria and the problems related to their monitoring and management in clinical practice.

Contribution 8 describe the epidemiology, molecular resistance and virulence features of KPC-*K. pneumoniae* strains collected from 2020 to 2023 (a period including the peak months of the COVID-19 pandemic) at the National Institute for Infectious Diseases Lazzaro Spallanzani IRCCS, which at that time was taking in an exceptional load of critically ill patients, having been designated the main COVID-19 hospital in the Lazio region. In view of the ECDC alert in 2021 [23] and concerned by the additional burden of dealing with the COVID-19 emergency, the authors decided to implement a hospital-wide genomic surveillance of carbapenem resistant-*K. pneumoniae* strains that included screening for virulence-associated genes. Strains collected in 2020 and the earlier months of 2021 (the peak of the COVID-19 period) were also retrospectively analyzed. It is known that hypervirulent *K. pneumoniae* (hvKp) are rarely MDR, but they show a high rate of occurrence in the community and a capability of infecting healthy individuals [24]; thus they may represent a serious health threat if combined with carbapenem resistance. Consistently, the results of the study document a single-event transfer of drug resistance genes and virulence genes, confirming the assumption that this type of monitoring is necessary.

Contribution 9 describes the epidemiology of Carbapenem-resistant Enterobacteriaceae (CRE) identified by multiplex RT-PCR in rectal swabs of patients upon admission to high-risk wards and compares data obtained from both molecular and culture CRE screening. In detail, rectal swabs, prospectively collected within 12–24 h of admission, underwent molecular screening for identification of *K. pneumoniae* carbapenemase (KPC), New Delhi metallo-β-lactamase (NDM), Verona integron-mediated metallo-β-lactamase (VIM), imipenemase (IMP), and OXA-48. Four percent of the samples were positive for at least one carbapenem-resistant gene by a molecular approach (MA), with KPC, NDM, and VIM having the highest prevalence. Culture testing confirmed the presence of carbapenemase in most samples but showed a disagreement rate of about 25% between the two methods, which, unfortunately, rose up to 60% for VIM. As a consequence, the authors strongly emphasize the value of molecular screening as a useful and fast method for the rapid identification of CRE genes among clinical isolates, making it possible to implement active surveillance.

Contribution 10 points out that while many studies have focused on CRE infections in adults, data on the prevalence and pathogenesis of *Klebsiella pneumoniae* carbapenemase (KPC)-producing *K. pneumoniae* (a type of CPE) in hospitalized children with hematological malignancies are scarce [25,26]. The authors show the results of an 8-year retrospective observational study on the effects of two therapeutic regimens against KPC-producing *Klebsiella pneumoniae* for Febrile Neutropenic Episodes (FNEs) in colonized children with Acute Leukemia (AL) infections, namely standard and active Empiric Antibiotic Treatment (EAT). Standard EAT included piperacillin–tazobactam with or without tigecycline. Active EAT included colistin combined with tigecycline and/or gentamicin. In children with AL, the mortality rate from *Klebsiella pneumoniae* carbapenemase (KPC)-producing *K. pneumoniae* bloodstream infection (KPC-KpBSI) is over 50% and highest when active treatment is delayed [27,28,29,30]. Neutropenic KPC-*K. pneumoniae* carriers are at a high risk of KPC-KpBSI, and pre-emptive Empiric Antibiotic Treatment of FNEs active against KPC-*K. pneumoniae* may reduce this mortality. Their findings strongly support the routine use of active EAT in febrile neutropenic children with Acute Leukemia who are KPC-*K. pneumoniae* carriers.

Always remaining in the pediatric field, Contribution 11 deals with urinary tract infections (UTIs), which are among the most common bacterial infections in children and constitute a frequent reason for medical evaluation, hospital admission and antibiotic prescription [31,32,33]. They affect especially infants and young children, in whom symptoms may be nonspecific so that diagnosis and therapy can often be delayed with serious consequences [34,35,36]. *Escherichia coli* (*E. coli*) is the primary etiologic agent of UTIs in children, responsible for approximately 80–90% of community-acquired cases, and recently a rising number of extended-spectrum β-lactamase (ESBL) producers, has made empirical treatment increasingly difficult [37]. In agreement with data available in the literature, this study, which assessed the antimicrobial susceptibility of *E. coli* strains isolated from pediatric UTI cases in a Romanian tertiary pediatric hospital over a three-year period (2022–2024), confirms a substantial percentage (19%) of ESBL-producing clinical isolates displaying high resistance to beta-lactams, including amoxicillin/clavulanic acid and cephalosporins, and reiterate the importance of routine local surveillance and updated antibiograms as essential to guide effective empirical therapy and limit the spread of AMR in children.

In relation to the UTIs, Contribution 12 reviews studies focused on the bacteriostatic/bactericidal activity, inhibition of bacterial adhesion and quorum sensing, restoration of uroepithelial integrity and immune response, by molecules, vitamins, and compounds obtained from plants. Although preliminary, such studies are very interesting especially in view of the necessity to find alternative and/or complementary therapies to counteract AMR.

Finally, the importance of continuous surveillance of local microbial ecology and its AMR patterns in hospital as well as community contexts is strongly reiterated by the results reported in Contribution 13, whose retrospective observational study analyzed respiratory microbial isolates collected from 2018 to 2023 in Tor Vergata University Hospital, Rome, Italy. The data were analyzed through WHOnet 2025 software, and the breakpoint references of EUCAST 2025 [38,39]. Analysis of more than 54,000 unique microorganism/drug associations, the majority of which were from inpatients (over 90%), confirmed the persistent and high prevalence and resistance to multiple antibiotics of *A. baumannii* and highlighted significant upward resistance trends of *K. pneumoniae* to multiple antibiotics, in accordance with previous local reports and the international literature [40,41,42,43]. Moreover, it showed that approximately 20% of clinical isolates were fungi, also including some non-albicans *Candida* (NAC) species, which exhibit intrinsic resistance to azoles. The increasing prevalence of fungi, particularly *C. Albicans*, during the pandemic years, align with the existing literature describing fungal overgrowth in critically ill and immunocompromised patients [44]. The study points out that although the local analysis showed low resistance rates versus antifungal molecules, the emergence of resistant NAC species and environmental fungi remains a growing concern, especially in hospital settings where antifungal susceptibility testing is performed only upon specific clinician requests.

All the contributions of this Special Issue acknowledge the importance of continuous, updated and systematic monitoring of the microorganisms responsible for hospital and community infections and their AMR patterns, which can only be the result of close collaboration between different and qualified professional skills. This cooperation, along with the findings emerging from basic research, including the discovery of new antibiotic molecules, implementation of vaccination policies and pursuing the One Health approach worldwide, will yield significant results in the fight against infectious diseases. This is our hope and wish for the new year in an age increasingly marked by conflicts and inequalities, reminding us that without others we cannot save neither ourselves nor our planet.
